# Design, Synthesis and In Vitro Mechanistic Investigation of Novel Hexacyclic Cage-Like Hybrid Heterocycles

**DOI:** 10.3390/molecules24213820

**Published:** 2019-10-23

**Authors:** Raju Suresh Kumar, Abdulrahman I. Almansour, Natarajan Arumugam, Faruq Mohammad

**Affiliations:** 1 Department of Chemistry, College of Science, King Saud University, P.O. Box 2455, Riyadh 11451, Saudi Arabia; almansor@ksu.edu.sa (A.I.A.); anatarajan@ksu.edu.sa (N.A.); 2 Surfactants Research Chair, Department of Chemistry, College of Science, King Saud University, P.O. Box 2455, Riyadh 11451, Saudi Arabia

**Keywords:** [3 + 2] cycloaddition, hexacyclic cage-like hybrid heterocycles, anticancer activity, in vitro toxicity, apoptotic mechanism, role of caspases

## Abstract

Novel hexacyclic cage-like hybrid heterocycles have been synthesized in excellent yields employing a relatively less explored non-stabilized azomethine ylides derived from acenaphthenequinone and tyrosine with functionalized dipolarophiles using [3 + 2] cycloaddition strategy. The synthesized hexacyclic cage-like hybrid heterocycles were characterized by spectroscopic analysis. Following the physical characterization, these cage-like hybrid heterocycles were tested for their biological activity by means of different cancer (A549 and Jurkat cells) and non-cancer (BRL-3A and PCS-130) in vitro cell culture systems. The results of the study under tested concentrations (up to 100 μM) indicated that these compounds are not affecting any viability to the cell growth of non-cancer cells, while providing significant anticancer activity against both of the cancer cells. Further analysis of in-depth mechanistic study for the cell death indicated that these compounds are exhibiting late apoptosis or early necrosis pathway to the cells where it is operated by the induction of caspases.

## 1. Introduction

Cancer is a disease that causes an abnormal growth of cells to the tissues, called a tumor, where the cells lose their normal functions and when it spreads to the other parts of the body (metastasis) that can eventually lead to death. The recent survey from the World Health Organization’s “Cancer Prevention and Control through an Integrated Approach” recognized the cancerous disease as the second foremost cause of morbidity which accounts for about 9.6 million deaths in its up-to-date statistics worldwide [1]. Also, the number of cancer diagnosed patients are increasing, as it was about 14.1 million during the year 2012 and is expected to be projected to 21.6 million by the year 2030 [2]. Hence, considering the significant increase in the number of cancer patients every year, the quest for the early diagnosis and safe treatment modalities are being developed by the researchers as the currently available methods are not up to their mark [3,4]. In general, for cancerous diseases, diagnosis at the earliest stage is the most important criteria in addition to directing the antitumor drugs to their targeted organs. The common mechanisms of antitumor agents that were used during cancer chemotherapy being the DNA cross-linking, alkylation, inhibition of DNA and RNA synthesis, inhibition of topoisomerase II functions and mitotic spindle formation and so forth. It was found in recent years that the apoptosis pathway also plays a key role in the degradation of cancer cells and in that way, a majority of antitumor drugs are being designed [5,6]. The one advantage of incorporating drugs with an apoptotic mechanism is that the cells undergo normal programmed cell death in order to maintain the tissue homeostasis as compared to the direct cell death, called necrosis. A majority of the antitumor drugs also show their toxicological activity to healthy normal tissues and in that way one can reduce the extent of side effects associated with the highly toxic chemotherapeutic drugs [7]. Also, the apoptotically dying cells are controlled by the cascade of events like initiator and effector caspases, cytotoxic T or NK cells which gets released sequentially under some physiological and pathological conditions of the cell wall. Furthermore, in the apoptotic pathway of cells, a type of proteolytic enzymes called the caspases which make up a family of cysteine proteases are to be released [8,9,10]. In that way for achieving the highest efficiency in any cancer chemotherapy, the regulation of caspases is highly important as the non-release of caspases may result in the drug-resisted tumor cells [11]. 

**Figure 1:**
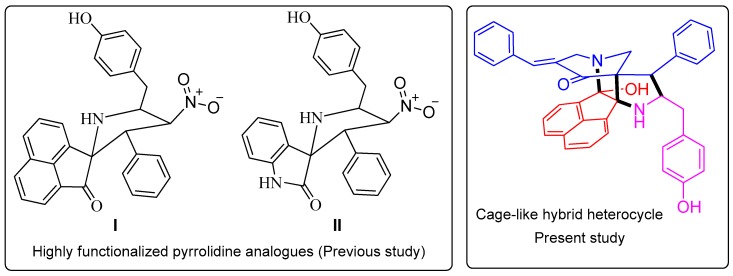
Schematic representation for the structural comparison of pyrrolidine analogues and cage-like hybrid heterocycles.

Some of the currently available cancer therapies like chemotherapy, radiation, surgery, gene therapy and so forth, suffer from the limitations of non-target associated side effects like hair loss, skin problems, nausea and vomiting, hormone imbalances and so forth, and this demands the development of novel anticancer agents with highest selectivity and negligible toxicity towards healthy tissues [12,13,14]. As a part of our research work directed towards the development of novel anticancer agents based on the ability to block cell proliferation and/or to induce apoptosis, we recently reported the synthesis and apoptotic inducing properties of highly functionalized pyrrolidine heterocyclic hybrids Figure 1 [15,16]. Some of the pyrrolidine analogues displayed promising activity and were found to promote apoptosis through the activation of caspase-3. We believed that it would be further informative to explore pyrrolidine integrated cage-like hybrid heterocycles in the quest of novel anticancer chemotherapeutics. The present work is also rooted in the fact that the naturally available cage-like compound, Gambogic acid, an effective antitumor agent that has recently passed phase IIa clinical trials, has antitumor activity that is considered to be due to the presence of α,β-unsaturated structural moiety and the peripheral moieties as suitable sites for diverse modification [17,18,19]. Therefore, keeping in mind the importance of pyrrolidine derivatives and α,β-unsaturated moiety, in the present study, the molecules are designed viz. the pyrrolidine assimilated cage-like hybrid heterocycles possessing the α,β-unsaturated moiety which appeared to be essential for the generation of antitumor activity. Hence, the construction of pyrrolidine integrated hexacyclic cage-like hybrid heterocycles employing [3 + 2] cycloaddition reaction of a relatively less explored azomethine ylide with functionalized dipolarophiles and their evaluation of cell viability towards cancer and non-cancer cells in addition to the mechanistic studies of toxicology is the subject of the present research article.

## 2. Results and Discussion

### 2.1. Chemistry

**Scheme 1:**
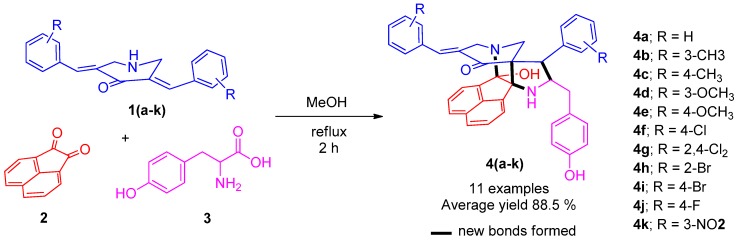
Synthesis of cage-like hybrid heterocycles **4**.

Our synthetic strategy comprises dipole generation, 1,3-cycloaddition and subsequent annulation steps to form the novel hexacyclic cage-like hybrid heterocycles. [3 + 2] Cycloaddition reaction of functionalized dipolarophiles viz, *N*-unsubstituted 3,5-bis[(*E*)-arylmethylidene]tetrahydro-4(1*H*)-pyridinones **1**(**a**–**k**) [20] with a relatively less explored azomethine ylide derived from acenaphthenequinone and tyrosine afforded the novel hexacyclic cage-like hybrid heterocycles. In a representative reaction, an equal molar ratio of **1b**, **2** and **3** in 10 mL of methanol in an oil bath was heated with constant stirring at 70 °C. The reaction progress was monitored after every 30 min interval by thin layer chromatography. After the reaction was completed (2 h), ice cold water (50 mL) was added to the reaction mixture. The precipitate obtained was filtered under vacuum and purified through crystallization using ethyl acetate. As estimated the reaction furnished the cage-like structure **4b** in an excellent yield (91%). Similarly, a series of dipolarophiles with different substituents on the aryl rings reacted with the azomethine ylides, all these reactions progressed efficiently in a highly regio- and stereocontrolled manner furnishing the cycloadducts in an excellent yields (83–92%). A total of eleven hexacyclic cage-like hybrid heterocycles **4**(**a**–**k**) were synthesized (Scheme 1).

**Figure 2:**
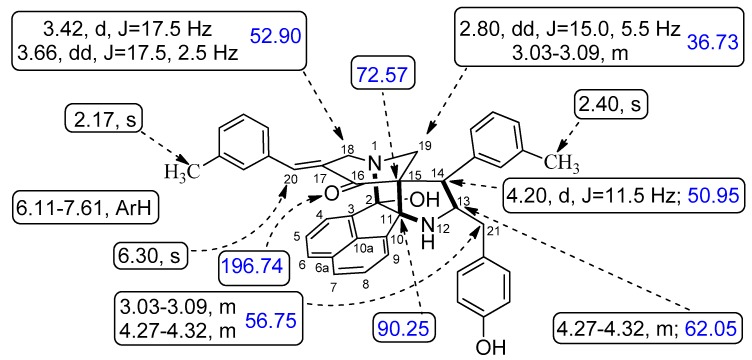
Arbitrary atom numbering and Selected ^1^H- and ^13^C-nuclear magnetic resonance (NMR) chemical shifts of **4b**.

Structural interpretation of all the eleven hexacyclic cage-like hybrid heterocycles **4**(**a**–**k**) was done by Fourier transform-infrared (FT-IR), nuclear magnetic resonance (NMR) spectroscopic and mass spectrometry data. As a representative case, the structural elucidation of **4b** is discussed. In the IR spectrum, the main infrared absorption peak at υ_max_ 3415 cm^−1^ is referred to N–H/O–H while the peaks at 1715 and 1690 cm^−1^ belongs to C = O and C = C respectively. In the ^1^H-NMR spectrum, H-14 proton appears as a doublet at 4.20 ppm (*J* = 11.5 Hz) and is further confirmed by its Heteronuclear Multiple Bond Correlation (HMBC) with the carbonyl group of piperidone ring C-16 at 196.74 ppm. H,H-Correlation Spectroscopy (H,H-COSY) of H-14 assigns the multiplet at 4.27–4.32 ppm to H-13. C,H-COSY correlation of H-14 and H-13 protons assigns the carbon signals at 50.95 and 62.05 ppm to C-14 and C-13 respectively. H,H-COSY correlation of H-13 ascribes the multiplets at 3.03-3.09 and 4.27-4.32 ppm to 21-CH_2_. The doublet at 3.42 ppm (*J* = 17.5 Hz) and a doublet of doublets at 3.66 ppm (*J* = 17.5, 2.5 Hz) is due to 18-CH_2_ protons while the other doublet of doublets at 2.80 ppm (*J* = 15.0, 5.5 Hz) and the multiplet at 3.03–3.09 ppm is assignable to 19-CH_2_ protons. The singlet at 6.30 is due to H-20 while the other singlets at 2.17 and 2.40 ppm is due to methyl protons of the *para*-methyl phenyl rings. The aromatic protons appear around 6.11-7.61 ppm as doublets and multiplets. Dept 135 and Heteronuclear Multiple Quantum Correlation (HMQC) spectra were used for assigning the carbon chemical shifts of remaining carbons. Figure 2 depicts the arbitrary atom numbering and selected ^1^H and ^13^C chemical shifts of **4b**. The structural assignment of other cage-like hybrid heterocycles was also performed by similar considerations.

**Scheme 2:**
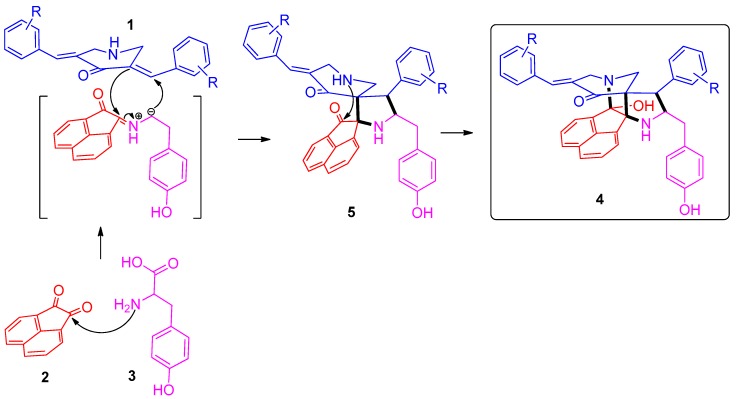
Probable mechanism for the formation of cage-like hybrid heterocycles **4**.

A reasonable mechanism for the assembly of cage-like hybrid heterocycles **4**(**a**–**k**) is consigned in Scheme 2 and is similar to the one as discussed in our earlier reports [21,22,23]. The less explored azomethine ylide generated in-situ from acenaphthenequinone **2** and tyrosine **3** adds to the dipolarophiles **1** from the less hindered bottom side affording the spiropyrrolidine intermediate **5**. Subsequent reaction of the amino function of the piperidone ring of the spiropyrrolidine intermediate **5** with the other carbonyl group of acenaphthenequinone results in the creation of cage-like hybrid heterocycles **4**.

### 2.2. Biology

**Figure 3:**
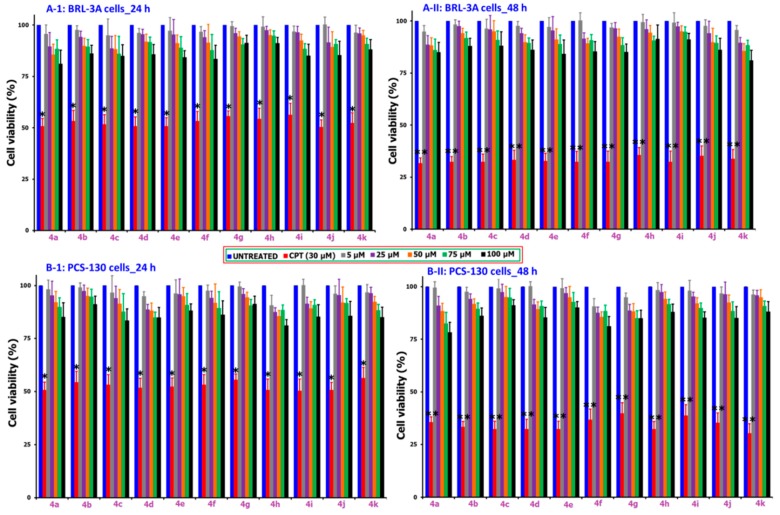
MTT assay comparison of BRL-3A and PCS-130 non-cancer cells at the two different intervals of 24 h and 48 h for the samples **4**(**a**–**k**) over a concentration range of 5–100 μM.

The MTT (3-(4,5-dimethylthiazol-2-yl)-2,5-diphenyl tetrazolium bromide) assay induced cell viability and proliferation studies for the compounds **4**(**a**–**k**) up to 100 μM concentration against the two non-cancer cell lines, BRL-3A (buffalo rat liver-3A) and PCS-130 (primary lung smooth muscle cells-130) and over the two time periods of 24 and 48 h were compared and are shown in Figure 3. From the comparison of results, it can be seen that the tested compounds up to 100 μM concentration are not toxic to either of the cell types as there is no significant loss in the viability of cells over the two time periods. However, under the same conditions of cell types and time periods, Camptothecin (CPT, 40 μM) which was used as the positive control, is indicated to offer a significant loss in the viability against both of the cell types and this loss found to be increased with an increase of incubation time. The observation of non-toxic behavior of our tested compounds **4**(**a**–**k**) against the non-cancer cell lines primarily providing the information that these compounds are very safe to use as there is no effect on the non-targeted cells. Such an observation of non-toxicity to the cells under the tested concentration can be attributed to the fact that the cells are resistant enough to fight against the toxic induced responses of the incubated compounds.

**Figure 4:**
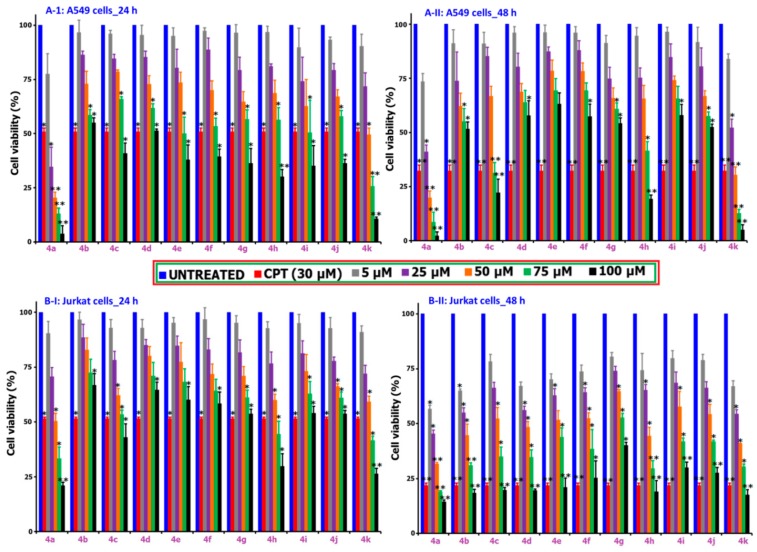
MTT assay comparison of A549 and Jurkat cells at the two different intervals of 24 h and 48 h for the samples **4**(**a**–**k**) over a concentration range of 5–100 μM.

The comparison of cell viability and proliferation studies of the testing compounds **4**(**a**–**k**) in the concentration range of 5–100 μM, time periods of 24 and 48 h against the two cancer cell lines of A549 and Jurkat cells are shown in Figure 4. The comparison and analysis of samples compound result against the positive control of CPT (40 μM), we observed that there is a significant loss in the viability of cells and this loss seem to be getting increased with an increase in the incubation period from 24 to 48 h. Based on the analysis of MTT assay results, the IC_50_ values were calculated for both of the cell types and the values are tabulated in Table 1. From the table, it can be observed that among the compounds **4**(**a**–**k**) tested, the two compounds **4a** and **4k** has significant activity and that too within those two, the compound **4a** has the highest activity as we observed by a very high loss in the viability to both of the cancer cells. In addition, we noticed that among the two different cancer cell lines tested, the response of A549 cells is very high against the Jurkat cells as we observed a greatest decrease in the viability of A549 cells and for that reason, we carried the further testings by taking the IC_50_ values of compound **4a** against the A549 cells. We also observed from Figure 4 that some of the compounds activity is better than the CPT’s activity and thereby providing the preliminary information that our synthesized compounds can serve as the better substituent to the CPT and its related compounds. Also, this type of behavior of compounds having different activities against various cell lines (as the cancer and non-cancer cells respond differently to our synthesized compounds) is expected and this can be attributed to the resistance offered by the intracellular proteins, that is, the cells are immune to the toxic-induced responses. 

The hexacyclic cage-like heterocyclic hybrids offer an intrinsic versatility and dynamic core scaffolding nature that can be very useful for the initial formation of bonding and associated cell death mechanisms that can finally lead the cells to death. Within the derivatives tested, the approximate activity follows the order of **4a** > **4k** > **4b** > **4g** > **4i** > **4h** > **4j** > **4e** > **4f** > **4d** > **4c**. Such a significant activity of compound **4a** when compared to other derivatives can be attributed to the solubility in cell culturing medium and associated increase in the interaction of **4a** with that of cells. Since **4a** maintains no extra substituted groups while all other derivatives contain either of –NO_2_, –CH_3_, –OCH_3_, –Cl, –Br and –F and some repetition of same groups with change of positions. In general, the hexacyclic cage-like heterocyclic hybrids maintain some inbuilt mechanism for the interaction with that of cells where they reduce the cell growth in a programmable manner. However, the presence of some extra groups either enhance or depress the activity of those heterocycles and in our case, they actually reduce the inbuilt activity. In a general way, the ‘Br’ substituted compound should have higher toxicological activity against the Cl, F groups and in our case the tested compounds are not following this order. The reason for this unusual activity cannot be known at this stage but can preliminarily be linked to the solubility and associated enhancement in the interaction and finally leads to the loss of cell viability. In a similar way, the other substituents like –CH_3_ and –OCH_3_ also reducing the activity due to their bulky nature and decreased solubility in the culturing medium. 

**Figure 5:**
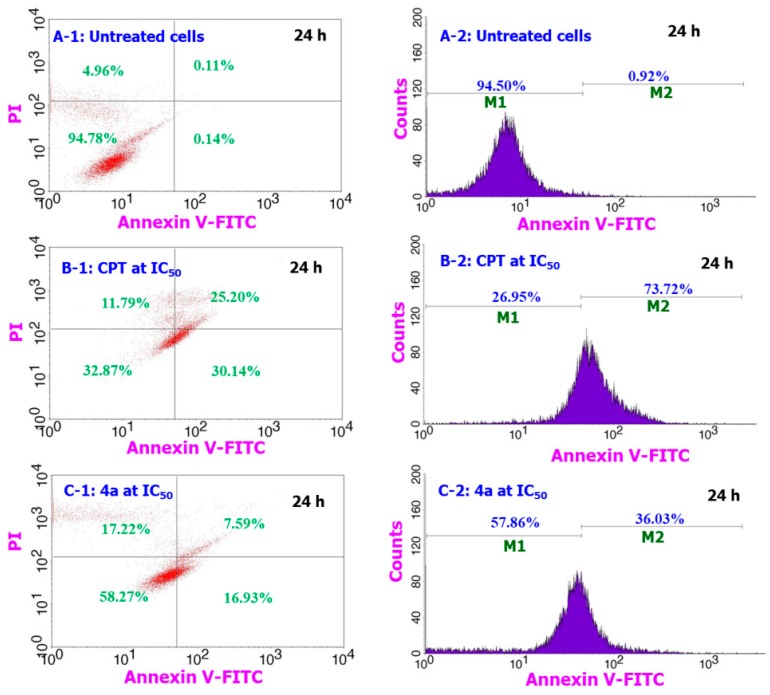
Comparison of apoptosis studies for the compound **4a** (**B**) with that of positive (**C**) and negative controls (**A**) for the A549 cells following the incubation period of 24 h.

**Figure 6:**
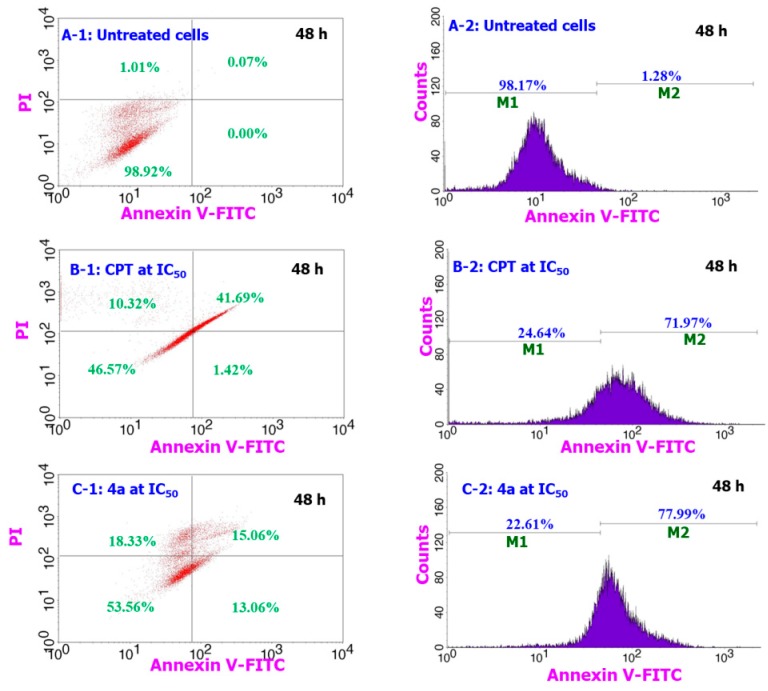
Comparison of apoptosis studies for the compound **4a** (**B**) with that of positive (**C**) and negative controls (**A**) for the A549 cells over the 48 h incubation period.

In order to investigate the mechanism of cell death induced by the highly active compound, the samples of **4a** at its IC_50_ concentration were tested against the A549 cells at two different time intervals of 24 and 48 h and the respective fluorescence intensities are shown in Figure 5 and Figure 6 respectively. From the analysis of results shown in Figure 5 of A-1, B-1 and C-1 that corresponds to the mixed fluorescence intensities from Annexin V-fluorescein isothiocyanate (Annexin V-FITC) and propidium iodide (PI) for the untreated, CPT and **4a** treated cells (respectively). It indicates from the results that there is an induction of apoptotic and necrotic pathway of mechanisms in the **4a** treated cells (as similar to CPT treated ones). In the case of 24 h cell treatment, we observed from Figure 5C-1 that about 7.5% of compound **4a** treated cells are undergoing the necrotic (or late apoptotic) pathway of cell death and this value is lower than the CPT treated ones (25% in Figure 5B-1). On further increase of incubation time to 48 h (Figure 6), we noticed that the compound **4a** treated cells are overtaking the CPT treated ones in terms of having the number of cells undergoing the necrotic (or late apoptotic) mechanism, that is, 15% for the CPT (Figure 6B-1) and 41.6% for the compound **4a** (Figure 6C-1). Such observation of highest necrotic or late apoptotic activity for the compound **4a** treated cells as compared against the CPT compound, confirms that the compound **4a** can be applied for the induction of cancer cell death in a late apoptotic or necrotic pathway. 

Similarly, from Figure 5 and Figure 6, M1 and M2 correspond to the Annexin V-FITC induced fluorescence intensities from the viable and apoptotically dying cells (respectively). The comparison of M1 and M2 values for the 24 h cell treatment (Figure 5), we noticed that the compound **4a** has a little lesser number (36%) of apoptotically dying cells (Figure 5C-2) as compared against the positive control CPT (73% as shown in Figure 5B-2). However, the same apoptotic cells for compound **4a** treated cells during the 48 h treatment (Figure 6C-2) are getting increased to 78% and leaving behind the CPT treated cells (72% as shown in Figure 6B-2). The observation of such an increase in the apoptotic activity of **4a** compound treated cells further confirms the evidence that the test sample **4a** is becoming more active during its 24–48 h of incubation time. 

**Figure 7:**
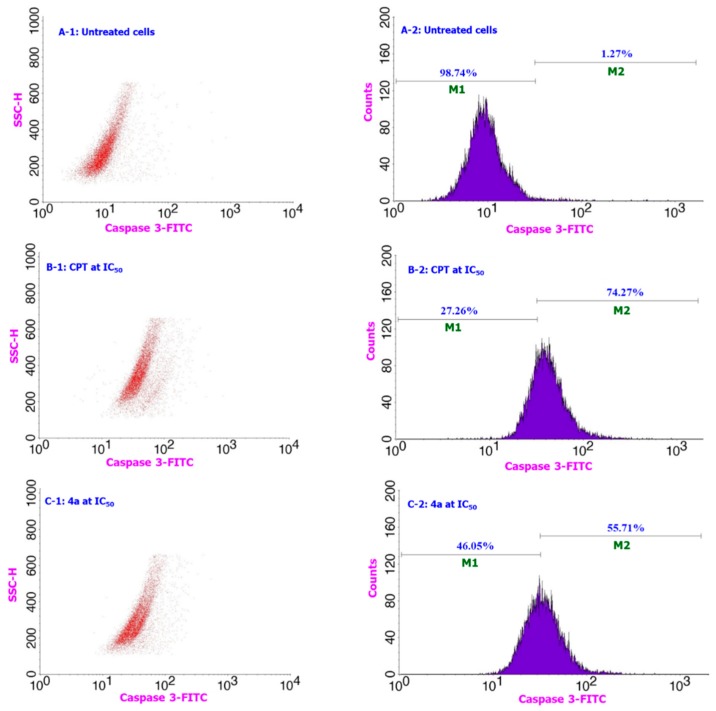
Comparison of compound **4a** (**C-1**,**C-2**) induced caspase-3 activity in A549 cells with that of positive (**B-1**,**B-2**) and negative (**A-1**,**A-2**) controls over a 24 h incubation period.

The FITC-induced fluorescence intensity to study the influence of caspase-3 in A549 cancer cells over a 24 h period for the compound **4a** were compared against the positive and negative controls where the results are shown in Figure 7. From the Figure 7C-2 showing the compound **4a** treated cells, about 55% of them are exhibiting the significant levels of fluorescence induced by the FITC dye as against the CPT of 74% (Figure 7B-2). These numbers seem to be quite low when we compare the results with that of negative control (cells of no treatment) as there is only 1.2% of cells (Figure 7A-2) exhibiting the fluorescence intensity and further supporting for the induction of caspase dependent pathway for compound **4a** treated cells. 

**Figure 8:**
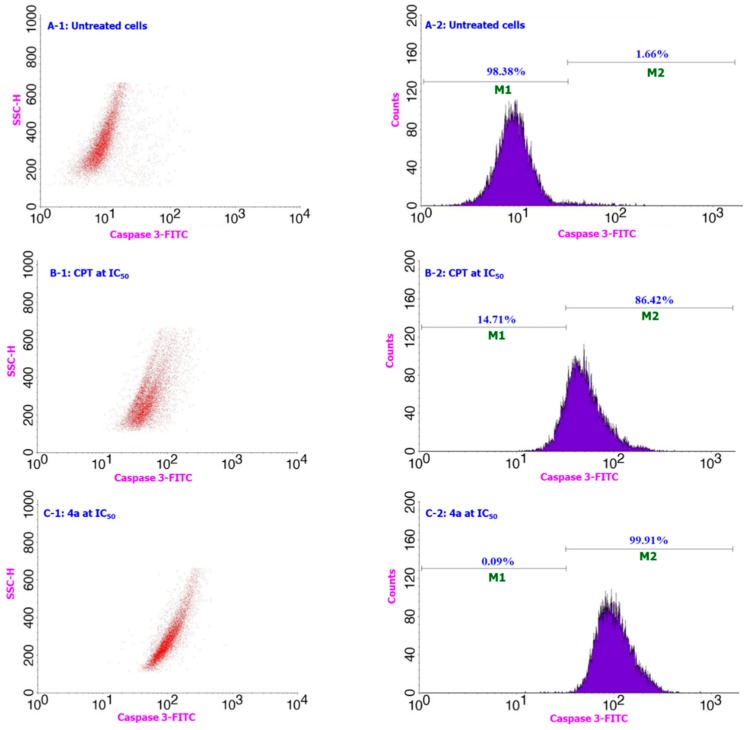
Comparison of compound **4a** (**C-1**,**C-2**) induced caspase-3 activity for the A549 cells with that of positive (**B-1**,**B-2**) and negative (**A-1**,**A-2**) controls over a 48 h incubation period.

**Figure 9:**
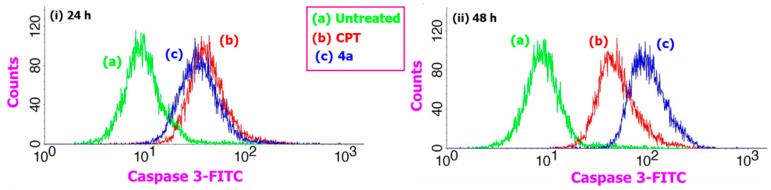
Overlay of fluorescence spectrums for the compound **4a** induced A549 cells with that of positive and negative controls during the two different intervals of 24 h (**i**) and 48 h (**ii**).

Similar studies of FITC-induced fluorescence activity to investigate the effect of caspase-3 for the compound **4a** treated cells over a 48 h period is shown in Figure 8. It can be observed from the figure that the number of cells undergoing the caspase-3 activity is highest for the compound **4a** treated cells, as we observed the fluorescence intensity in about 99.9% cells (Figure 8C-2), leaving behind the CPT treated cells with 86.4% (Figure 8B-2) and untreated cells with 1.6% (Figure 8A-2). Further, the overlay of same fluorescence spectrums from the caspase-3 dependent activity for the two time periods of 24 and 48 h are shown in Figure 9i,ii. The comparison of fluorescence intensities provides the clear picture how the lesser activity of compound **4a** during the 24 h incubation period (Figure 9i) is getting switched to the more dominant side (quite higher than the CPT) during its 48 h treatment (Figure 9ii).This provides the information for the increased cell viability losses on increasing the incubation time as there is only a moderate amount of caspases that are getting participated in the cell death during the first 24 h period and on continuing the incubation period to 48 h, almost all the cells are undergoing the caspase-induced pathway. The observation of such an exclusive cell death pathway for the compound **4a** treated cells confirms the occurrence of apoptotic type of programmed cell death. 

From the cumulative analysis of results discussed in MTT, apoptotic and caspase activity assays, it is inferred that the compound **4a** is exhibiting the highest activity against both of the cancer cell types and no significant activity against the non-cancer cells. Within the two cancer cells, the compound **4a** is highly active against the A549 cells as against the Jurkat cells and this activity is time dependent as we observed an increase in the viability losses on incubation of cells from 24 to 48 h. Further analysis of cell death mechanism as compared against the CPT indicated that the compound **4a** is inducing the late apoptosis to the A549 cells and this mechanism is operated by the caspases. We also noticed that the apoptosis mechanism or the caspase activity is becoming more significant only after the 24 h incubation time (or during 24–48 h time period). The results generated in this study can be used for the development of new cancer treatment strategies with no or minimal side effects to the non-cancer cells and that too for the cancer cells, the programmed way of cell death can be induced.

## 3. Materials and Methods

### 3.1. Chemistry

General Procedure for The Synthesis of Cage-Like Hybrid Heterocycles **4**(**a**–**k**)

An equal mole ratio of **1**(**a**–**k**), **2** and **3** were dissolved in MeOH (15 mL) and heated under reflux in an oil bath for 2 h. After completion of the reaction as evident from TLC, the reaction mixture was kept aside at room temperature overnight. The precipitate obtained was filtered and dried under vacuum.

#### 3.1.1. Hexacyclic Cage-Like Hybrid Heterocycle (**4a**) 

Pale yellow solid, 89% yield; mp 189–191 °C; IR (KBr) ν_max_ 3421, 1718, 1694 cm^−1^; ^1^H-NMR (500 MHz, CDCl_3_): δ_H_ 2.76 (1H, d, *J* = 14.5 Hz, 19-CH_2_), 3.01–3.06 (2H, m, 19-CH_2_ and 21-CH_2_), 3.41 (1H, d, *J* = 17.5 Hz, 18-CH_2_), 3.68 (1H, d, *J* = 17.5 Hz, 18-CH_2_), 4.16-4.30 (3H, m, H-13, H-14 and 21-CH_2_), 6.30–6.51 (4H, m, Ar–H), 6.86–7.07 (6H, m, Ar–H), 7.25–7.37 (6H, m, Ar–H), 7.44 (1H, d, *J* = 7.5 Hz, Ar–H), 7.51 (1H, d, *J* = 6.0 Hz, Ar–H), 7.55–7.63 (3H, m, Ar–H). ^13^C-NMR (125 MHz, CDCl_3_): δ_C_ 36.57, 50.87, 52.82, 56.61, 61.99, 72.32, 90.17, 103.68, 115.88, 120.96, 121.34, 125.51, 125.73, 127.30, 127.76, 128.42, 128.46, 128.57, 128.71, 128.97, 129.46, 130.28, 130.47, 131.05, 132.51, 133.72, 136.00, 136.26, 136.62, 138.47, 140.59, 155.62, 196.74. LC/MS(ESI): *m/z* = 576 (M^+^). Anal. calcd. for C_39_H_32_N_2_O_3_: C, 81.23; H, 5.59; N, 4.86. Found: C, 81.39; H, 5.50; N, 4.69%.

#### 3.1.2. Hexacyclic Cage-Like Hybrid Heterocycle (**4b**) 

Pale yellow solid, 91% yield; mp 166–168 °C; IR (KBr) ν_max_ 3415, 1715, 1690 cm^−1^; ^1^H-NMR (500 MHz, CDCl_3_): δ_H_ 2.17 (3H, s, CH_3_), 2.40 (3H, s, CH_3_), 2.80 (1H, dd, *J* = 15.0, 5.5 Hz, 19-CH_2_), 3.03-3.09 (2H, m, 19-CH_2_ and 21-CH_2_), 3.42 (1H, d, *J* = 17.5 Hz, 18-CH_2_), 3.66 (1H, dd, *J* = 17.5, 2.5 Hz, 18-CH_2_), 4.20 (1H, d, *J* = 11.5 Hz, H-14), 4.27-4.32 (2H, m, H-13 and 21-CH_2_), 6.08–6.15 (1H, m, Ar–H), 6.19 (1H, d, *J* = 7.5 Hz, Ar–H), 6.30 (1H, s, H-20), 6.53 (1H, d, *J* = 7.0 Hz, Ar–H), 6.85 (2H, d, *J* = 8.0 Hz, Ar–H), 6.89–6.95 (2H, m, Ar–H), 7.04 (2H, d, *J* = 8.0 Hz, Ar–H), 7.11 (1H, d, *J* = 7.5 Hz, Ar–H), 7.27–7.41 (5H, m, Ar–H), 7.49 (1H, d, *J* = 8.0 Hz, Ar–H), 7.54 (1H, d, *J* = 7.0 Hz, Ar–H), 7.60 (1H, d, *J* = 8.0 Hz, Ar–H). ^13^C-NMR (125 MHz, CDCl_3_): δ_C_ 21.18, 21.64, 36.73, 50.95, 52.90, 56.75, 62.05, 72.57, 90.25, 103.74, 115.75, 120.91, 121.34, 125.41, 125.70, 125.75, 126.32, 127.64, 127.87, 127.95, 128.06, 128.40, 128.54, 129.15, 130.02, 130.16, 130.41, 131.11, 132.60, 133.84, 136.06, 136.41, 136.60, 137.30, 138.24, 138.73, 140.84, 155.13, 196.74. LC/MS(ESI): *m/z* = 604 (M^+^). Anal. calcd. for C_41_H_36_N_2_O_3_: C, 81.43; H, 6.00; N, 4.63. Found: C, 81.57; H, 6.09; N, 4.41%.

#### 3.1.3. Hexacyclic Cage-Like Hybrid Heterocycle (**4c**)

Pale yellow solid, 90% yield; mp 175–177 °C; IR (KBr) ν_max_ 3417, 1720, 1692 cm^−1^; ^1^H-NMR (500 MHz, CDCl_3_): δ_H_ 2.20 (3H, s, CH_3_), 2.34 (3H, s, CH_3_), 2.77 (1H, dd, *J* = 15.0, 5.0 Hz, 19-CH_2_), 3.00 (1H, d, *J* = 12.5 Hz, 21-CH_2_), 3.04-3.07 (1H, m, 19-CH_2_), 3.41 (1H, d, *J* = 17.5 Hz, 18-CH_2_), 3.69 (1H, dd, *J* = 17.5 Hz, 18-CH_2_), 4.16–4.27 (3H, m, H-14, H-13 and 21-CH_2_), 6.26–6.35 (3H, m, Ar–H), 6.49 (1H, d, *J* = 6.5 Hz, Ar–H), 6.81 (2H, d, *J* = 8.0 Hz, Ar–H), 6.85 (2H, d, *J* = 8.0 Hz, Ar–H), 7.01 (2H, d, *J* = 8.0 Hz, Ar–H), 7.18-7.34 (4H, m, Ar–H), 7.45–7.57 (5H, m, Ar–H). ^13^C-NMR (125 MHz, CDCl_3_): δ_C_ 21.04, 21.25, 36.63, 50.58, 52.94, 56.60, 62.00, 72.45, 90.18, 103.73, 115.75, 120.92, 121.29, 125.48, 125.66, 127.47, 127.85, 127.95, 128.37, 128.58, 128.84, 129.40, 129.71, 130.33, 130.62, 131.11, 131.74, 133.62, 135.31, 136.28, 136.89, 138.75, 140.83, 155.14, 196.74. LC/MS(ESI): *m/z* = 604 (M^+^). Anal. calcd. for C_41_H_36_N_2_O_3_: C, 81.43; H, 6.00; N, 4.63. Found: C, 81.61; H, 6.16; N, 4.74%.

#### 3.1.4. Hexacyclic Cage-Like Hybrid Heterocycle (**4d**)

Yellow solid, 85% yield; mp 201–203 °C; IR (KBr) ν_max_ 3421, 1718, 1694 cm^−1^; ^1^H-NMR (500 MHz, CDCl_3_): δ_H_ 2.80 (1H, dd, *J* = 14.5, 5.0 Hz, 19-CH_2_), 3.06-3.10 (2H, m, 19-CH_2_ and21-CH_2_), 3.45 (1H, d, *J* = 17.0 Hz, 18-CH_2_), 3.66 (3H, s, OCH_3_), 3.76 (1H, dd, *J* = 17.0 Hz, 18-CH_2_), 3.82 (3H, s, OCH_3_), 4.21 (1H, d, *J* = 11.5 Hz, H-14), 4.27-4.36 (2H, m, H-13 and 21-CH_2_), 5.90 (1H, s, Ar–H), 5.97 (1H, d, *J* = 7.0 Hz, Ar–H), 6.33 (1H, s, Ar–H), 6.53 (1H, d, *J* = 7.0 Hz, Ar–H), 6.66 (1H, dd, *J* = 8.5, 2.5 Hz, Ar–H), 6.82–6.85 (3H, m, Ar–H), 6.95–7.03 (3H, m, Ar–H), 7.12–7.15 (2H, m, Ar–H), 7.29–7.40 (3H, m, Ar–H), 7.51 (1H, d, *J* = 8.0 Hz, Ar–H), 7.60–7.63 (2H, m, Ar–H). ^13^C-NMR (125 MHz, CDCl_3_): δ_C_ 36.83, 50.79, 52.80, 55.23, 55.36, 56.61, 62.01, 72.50, 90.01, 104.69, 110.46, 112.85, 114.94, 115.28, 115.88,118.98, 120.91, 121.90, 125.94, 127.57, 127.92, 128.07, 128.63, 129.02, 129.82, 130.41, 131.18, 133.54, 134.90, 135.41, 136.11, 137.92, 140.75, 155.23, 158.98, 159.89, 196.81. LC/MS(ESI): *m/z* = 636 (M^+^). Anal. calcd. for C_41_H_36_N_2_O_5_: C, 77.34; H, 5.70; N, 4.40. Found: C, 77.52; H, 5.54; N, 4.51%.

#### 3.1.5. Hexacyclic Cage-Like Hybrid Heterocycle (**4e**)

Yellow solid, 83% yield; mp 190–192 °C; IR (KBr) ν_max_ 3424, 1715, 1692 cm^−1^; ^1^H-NMR (500 MHz, CDCl_3_): δ_H_ 2.78 (1H, dd, *J* = 15.0, 4.5 Hz, 19-CH_2_), 3.01–3.08 (2H, m, 19-CH_2_ and 21-CH_2_), 3.41 (1H, d, *J* = 17.0 Hz, 18-CH_2_), 3.72 (3H, s, OCH_3_), 3.75-3.78 (1H, m, 18-CH_2_), 3.81 (3H, s, OCH_3_), 4.19–4.26 (3H, m, H-14, H-13 and 21-CH_2_), 6.32 (1H, s, Ar–H), 6.45 (2H, d, *J* = 8.5 Hz, Ar–H), 6.51 (1H, d, *J* = 7.0 Hz, Ar–H), 6.59 (2H, d, *J* = 9.0 Hz, Ar–H), 6.82 (2H, d, *J* = 8.0 Hz, Ar–H), 6.92 (2H, d, *J* = 8.5 Hz, Ar–H), 7.02 (2H, d, *J* = 8.0 Hz, Ar–H), 7.29–7.35 (2H, m, Ar–H), 7.46–7.57 (4H, m, Ar–H), 7.81–7.85 (1H, m, Ar–H). ^13^C-NMR (125 MHz, CDCl_3_): δ_C_ 36.68, 50.16, 52.84, 55.20, 55.26, 56.64, 62.03, 72.28, 90.04, 103.83, 113.39, 114.12, 115.75, 120.91, 121.31, 125.51, 125.62, 126.64, 127.48, 127.84, 127.97, 128.37, 128.63, 129.92, 130.29, 131.08, 131.71, 133.45, 135.32, 136.02, 140.72, 155.16, 158.74, 159.83, 196.78. LC/MS(ESI): *m/z* = 636 (M^+^). Anal. calcd. for C_41_H_36_N_2_O_5_: C, 77.34; H, 5.70; N, 4.40. Found: C, 77.47; H, 5.85; N, 4.25%.

#### 3.1.6. Hexacyclic Cage-Like Hybrid Heterocycle (**4f**)

White solid, 92% yield; mp 170–172°C; IR (KBr) ν_max_ 3420, 1716, 1695 cm^−1^; ^1^H-NMR (500 MHz, CDCl_3_): δ_H_ 2.79 (1H, dd, *J* = 15.0, 5.0 Hz, 19-CH_2_), 3.00 (1H, d, *J* = 13.0 Hz, 21-CH_2_), 3.06 (1H, dd, *J* = 15.0, 4.0 Hz, 19-CH_2_), 3.36 (1H, d, *J* = 17.5 Hz, 18-CH_2_), 3.66 (1H, dd, *J* = 17.5, 2.0 Hz, 18-CH_2_), 4.18–4.26 (3H, m, H-14, H-13 and 21-CH_2_), 6.22 (1H, s, Ar–H), 6.29 (2H, d, *J* = 8.0 Hz, Ar–H), 6.50 (1H, d, *J* = 7.0 Hz, Ar–H), 6.86 (2H, d, *J* = 8.0 Hz, Ar–H), 6.99–7.04 (4H, m, Ar–H), 7.30–7.39 (4H, m, Ar–H), 7.49–7.55 (4H, m, Ar–H), 7.60 (1H, d, *J* = 8.5 Hz, Ar–H). ^13^C-NMR (125 MHz, CDCl_3_): δ_C_ 36.61, 50.29, 52.87, 56.61, 61.94, 72.41, 90.19, 103.75, 115.86, 120.91, 121.46, 125.58, 125.83, 127.45, 128.03, 128.12, 128.45, 128.92, 130.27, 130.68, 131.07, 132.14, 133.16, 133.22, 134.52, 134.78, 135.10, 135.99, 138.48, 140.54, 155.37, 196.54. LC/MS(ESI): *m/z* = 645 (M^+^). Anal. calcd. for C_39_H_30_Cl_2_N_2_O_3_: C, 72.56; H, 4.68; N, 4.34. Found: C, 72.78; H, 4.53; N, 4.48%.

#### 3.1.7. Hexacyclic Cage-Like Hybrid Heterocycle (**4g**) 

Pale yellow solid, 88% yield; mp 186–188 °C; IR (KBr) ν_max_ 3425, 1718, 1690 cm^−1^; ^1^H-NMR (500 MHz, CDCl_3_): δ_H_ 2.77 (1H, dd, *J* = 15.0, 6.0 Hz, 19-CH_2_), 3.01 (1H, dd, *J* = 15.0, 4.5 Hz, 19-CH_2_), 3.25 (1H, d, *J* = 12.0 Hz, 21-CH_2_), 3.38 (1H, d, *J* = 17.5 Hz, 18-CH_2_), 3.67 (1H, dd, *J* = 17.5, 3.0 Hz, 18-CH_2_), 4.01–4.08 (1H, m, H-14),4.55 (1H, d, *J* = 11.5 Hz, 21-CH_2_), 4.78 (1H, d, *J* = 10.5 Hz, H-13), 5.92 (1H, d, *J* = 8.5 Hz, Ar–H), 6.45 (1H, s, Ar–H), 6.74 (2H, d, *J* = 8.5 Hz, Ar–H), 6.91 (1H, dd, *J* = 8.0, 1.5 Hz, Ar–H), 7.00 (1H, d, *J* = 8.0 Hz, Ar–H), 7.06 (2H, d, *J* = 8.5 Hz, Ar–H), 7.13 (1H, d, *J* = 1.5 Hz, Ar–H), 7.23–7.29 (2H, m, Ar–H), 7.42-7.49 (2H, m, Ar–H), 7.53 (1H, d, *J* = 2.0 Hz, Ar–H), 7.55 (1H, d, *J* = 6.5 Hz, Ar–H), 7.65 (1H, d, *J* = 8.0 Hz, Ar–H), 7.70 (1H, d, *J* = 8.5 Hz, Ar–H). ^13^C-NMR (125 MHz, CDCl_3_): δ_C_ 38.20, 50.85, 52.30, 57.20, 64.80, 72.89, 90.35, 103.70, 115.61, 121.20, 121.46, 122.11, 125.81, 126.30, 126.49, 127.11, 128.16, 128.46, 128.71, 129.16, 129.91, 130.32, 130.55, 130.68, 130.82, 131.64, 132.64, 133.30, 133.63, 134.32, 134.72, 134.80, 136.07, 138.44, 140.08, 154.88, 195.18. LC/MS(ESI): *m/z* = 714 (M^+^). Anal. calcd. for C_39_H_28_Cl_4_N_2_O_3_: C, 65.56; H, 3.95; N, 3.92. Found: C, 65.74; H, 3.78; N, 3.83%.

#### 3.1.8. Hexacyclic Cage-Like Hybrid Heterocycle (**4h**)

Pale yellow solid, 86% yield; mp 183–185 °C; IR (KBr) ν_max_ 3421, 1714, 1690 cm^−1^; ^1^H-NMR (500 MHz, CDCl_3_): δ_H_ 2.78 (1H, dd, *J* = 15.0, 7.5 Hz, 19-CH_2_), 3.06 (1H, dd, *J* = 15.0, 3.5 Hz, 19-CH_2_), 3.32 (1H, d, *J* = 12.0 Hz, 21-CH_2_), 3.43 (1H, d, *J* = 17.5 Hz, 18-CH_2_), 3.68 (1H, dd, *J* = 18.5, 3.0 Hz, 18-CH_2_), 3.97-4.06 (1H, m, H-14), 4.59 (1H, d, *J* = 12.0 Hz, 21-CH_2_), 4.90 (1H, d, *J* = 10.5 Hz, H-13),5.85-5.87 (1H, m, Ar–H), 6.56–6.59 (1H, m, Ar–H), 6.72 (2H, d, *J* = 8.5 Hz, Ar–H), 6.93-6.96 (2H, m, Ar–H), 7.06 (2H, d, *J* = 8.5 Hz, Ar–H), 7.12–7.15 (1H, m, Ar–H), 7.23–7.34 (4H, m, Ar–H), 7.41–7.44 (1H, m, Ar–H), 7.50–7.56 (2H, m, Ar–H), 7.67–7.74 (3H, m, Ar–H). ^13^C-NMR (125 MHz, CDCl_3_): δ_C_ 38.97, 52.03, 52.35, 57.16, 65.60, 73.01, 90.46, 103.71, 115.55, 121.27, 121.46, 123.71, 125.85, 126.47, 126.54, 127.33, 127.45, 127.69, 128.15, 128.72, 128.76, 128.93, 129.10, 129.59, 130.18, 130.72, 132.46, 133.47, 134.33, 135.21, 135.33, 136.16, 136.40, 138.62, 140.42, 154.76, 195.20. LC/MS(ESI): *m/z* = 734 (M^+^). Anal. calcd. for C_39_H_30_Br_2_NO: C, 63.78; H, 4.12; N, 3.81. Found: C, 63.55; H, 4.23; N, 3.67%.

#### 3.1.9. Hexacyclic Cage-Like Hybrid Heterocycle (**4i**)

Pale yellow solid, 90% yield; mp 187–189 °C; IR (KBr) ν_max_ 3427, 1721, 1685 cm^−1^; ^1^H-NMR (500 MHz, CDCl_3_): δ_H_ 2.79 (1H, dd, *J* = 15.0, 5.0 Hz, 19-CH_2_), 2.99 (1H, d, *J* = 12.5 Hz, 21-CH_2_), 3.06 (1H, dd, *J* = 14.5, 4.0 Hz, 19-CH_2_), 3.35 (1H, d, *J* = 17.5 Hz, 18-CH_2_), 3.66 (1H, d, *J* = 17.5 Hz, 18-CH_2_), 4.15-4.26 (3H, m, H-14, H-13 and 21-CH_2_), 6.20–6.24 (3H, m, Ar–H), 6.49 (1H, d, *J* = 7.0 Hz, Ar–H), 6.81 (2H, d, *J* = 8.0 Hz, Ar–H), 7.00 (2H, d, *J* = 8.0 Hz, Ar–H), 7.18–7.38 (6H, m, Ar–H), 7.44 (2H, d, *J* = 9.0 Hz, Ar–H), 7.55 (2H, d, *J* = 7.0 Hz, Ar–H), 7.62 (1H, d, *J* = 8.5 Hz, Ar–H). ^13^C-NMR (125 MHz, CDCl_3_): δ_C_ 36.34, 50.33, 52.74, 55.94, 61.84, 71.49, 90.20, 103.09, 115.82, 120.85, 121.40, 125.64, 125.75, 127.57, 128.00, 128.20, 128.49, 128.84, 130.25, 130.62, 131.09, 132.28, 133.15, 133.26, 134.50, 135.18, 135.90, 138.57, 140.51, 154.94, 196.10. LC/MS(ESI): *m/z* = 734 (M^+^). Anal. calcd. for C_39_H_30_Br_2_NO: C, 63.78; H, 4.12; N, 3.81. Found: C, 63.91; H, 4.27; N, 3.95%.

#### 3.1.10. Hexacyclic Cage-Like Hybrid Heterocycle (**4j**)

White solid, 89% yield; mp 176–178 °C; IR (KBr) ν_max_ 3432, 1716, 1691 cm^−1^; ^1^H-NMR (500 MHz, CDCl_3_): δ_H_ 2.79 (1H, dd, *J* = 14.5, 4.0 Hz, 19-CH_2_), 2.99 (1H, d, *J* = 12.5 Hz, 21-CH_2_), 3.06 (1H, dd, *J* = 14.0, 3.5 Hz, 19-CH_2_), 3.36 (1H, d, *J* = 17.5 Hz, 18-CH_2_), 3.67 (1H, d, *J* = 17.5 Hz, 18-CH_2_), 4.18–4.25 (3H, m, H-14, H-13 and 21-CH_2_), 6.22 (1H, s, Ar–H), 6.36–6.38 (2H, m, Ar–H), 6.51 (1H, d, *J* = 7.0 Hz, Ar–H), 6.74-6.77 (2H, m, Ar–H), 6.83 (2H, d, *J* = 8.0 Hz, Ar–H), 7.01 (2H, d, *J* = 8.0 Hz, Ar–H), 7.06–7.09 (2H, m, Ar–H), 7.29–7.38 (3H, m, Ar–H), 7.48 (1H, d, *J* = 8.0 Hz, Ar–H), 7.52–7.54 (2H, m, Ar–H), 7.60 (1H, d, *J* = 8.0 Hz, Ar–H). ^13^C-NMR (125 MHz, CDCl_3_): δ_C_ 36.66, 50.22, 52.85, 56.74, 62.09, 72.40, 90.18, 103.73, 114.95, 115.59, 115.76, 120.83, 121.37, 125.46, 125.74, 127.79, 127.92, 128.39, 129.92, 130.30, 130.42, 131.07, 131.46, 132.32, 132.68, 134.84, 136.01, 138.67, 140.74, 155.09, 162.03, 162.38, 196.77. LC/MS(ESI): *m/z* = 612 (M^+^). Anal. calcd. for C_39_H_30_F_2_N_2_O_3_: C, 76.46; H, 4.94; N, 4.57. Found: C, 76.67; H, 4.80; N, 4.49%.

#### 3.1.11. Hexacyclic Cage-Like Hybrid Heterocycle (**4k**)

Pale yellow solid, 91% yield; mp 194–196 °C; IR (KBr) ν_max_ 3422, 1714, 1695 cm^−1^; ^1^H-NMR (500 MHz, CDCl_3_): δ_H_ 2.88 (1H, dd, *J* = 14.5, 5.0 Hz, 19-CH_2_), 3.00 (1H, d, *J* = 12.0 Hz, 21-CH_2_), 3.07 (1H, dd, *J* = 14.5, 4.5 Hz, 19-CH_2_), 3.29 (1H, d, *J* = 17.5 Hz, 18-CH_2_), 3.65 (1H, dd, *J* = 17.5, 2.5 Hz, 18-CH_2_), 4.29-4.38 (3H, m, H-14, H-13 and 21-CH_2_), 6.18 (1H, s, Ar–H), 6.61 (1H, d, *J* = 7.0 Hz, Ar–H), 6.65 (1H, d, *J* = 7.5 Hz, Ar–H), 6.82 (2H, d, *J* = 8.5 Hz, Ar–H), 7.01 (2H, d, *J* = 8.0 Hz, Ar–H), 7.05 (1H, s, Ar–H), 7.23-7.25 (2H, m, Ar–H), 7.46-7.56 (4H, m, Ar–H), 7.74 (1H, d, *J* = 8.0 Hz, Ar–H), 7.91 (1H, d, *J* = 8.0 Hz, Ar–H), 7.96 (1H, dd, *J* = 8.0, 1.5 Hz, Ar–H), 8.16 (1H, dd, *J* = 9.5, 1.5 Hz, Ar–H),8.42 (1H, s, Ar–H). ^13^C-NMR (125 MHz, CDCl_3_): δ_C_ 37.09, 50.88, 52.92, 56.89, 62.06, 72.62, 90.37, 103.78, 115.84, 120.93, 121.54, 122.44, 122.91, 123.73, 123.98, 125.53, 126.18, 127.32, 128.03, 128.68, 128.81, 129.68, 130.41, 131.04, 132.97, 134.35, 135.17, 135.20, 135.78, 135.95, 138.39, 138.88, 140.32, 147.57, 148.02, 155.09, 196.40. LC/MS(ESI): *m/z* = 666 (M^+^). Anal. calcd. for C_39_H_30_N_4_O_7_: C, 70.26; H, 4.54; N, 8.40. Found: C, 70.39; H, 4.73; N, 8.23%.

### 3.2. Biology

Detailed methodology has been provided in the Supplementary Section.

## 4. Conclusions

In conclusion, a series of novel hexacyclic cage-like hybrid heterocycles with different substitutions on the aryl rings have been synthesized in good to excellent yields using [3 + 2] cycloaddition strategy employing a relatively less explored non-stabilized azomethine ylides derived from acenaphthenequinone and tyrosine with functionalized dipolarophiles. The biological activity studies of these synthesized compounds revealed that they have no activity against the healthy normal cells under the tested concentrations, while significant anti-cancer activity against the cancer cell types. Among all the derivatives, the less substituted compound found to have the significant anti-cancer activity as against the other derivatives and this can be attributed to the enhanced solubility and associated interaction of the compound with that of cells. In addition, the result of mechanistic analysis of cell death indicated that the cells are experiencing late apoptotic or early necrosis pathway which is operated by the caspases. Furthermore, the results of present study provide the experimental proof for treating the cancer cells with minimal or no damage to the healthy normal cells.

## Figures and Tables

**Table 1:** Comparison of the IC_50_ concentrations for compounds **4**(**a**–**k**) against A549 and Jurkat cells.

Entry	Compound	IC_50_ Values (µM, 24 h)		IC_50_ Values (µM, 48 h)	
		A549	Jurkat	A549	Jurkat
1	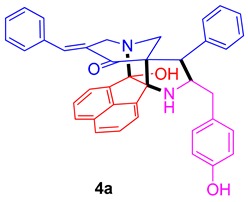	21.89 ± 2.1	24.8 ± 3.1	14.9 ± 3.0	23.8 ± 2.8
2	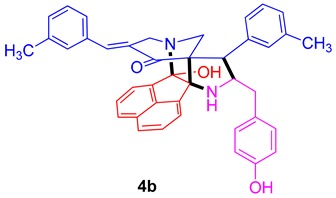	65.45 ± 4.9	55.5 ± 4.4	44.7 ± 3.7	36.2 ± 4.2
3	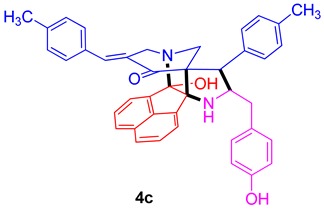	93.08 ± 7.1	91.9 ± 6.8	58.9 ± 3.7	51.4 ± 4.6
4	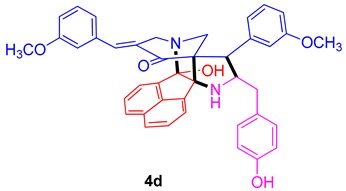	83.78 ± 4.7	85.4 ± 4.8	74.6 ± 4.1	41 ± 3.4
5	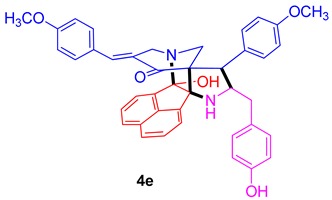	71.6 ± 3.6	82.2 ± 2.5	70.8 ± 2.2	51 ± 6.8
6	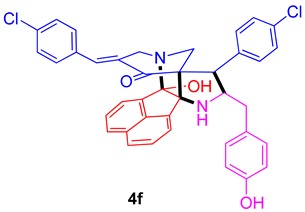	72.6 ± 4.1	90.4 ± 2.4	65 ± 2.6	53.4 ± 8.9
7	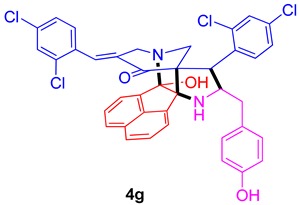	66.2 ± 3.5	73.7 ± 4.4	59.1 ± 3.4	80.1 ± 3.5
8	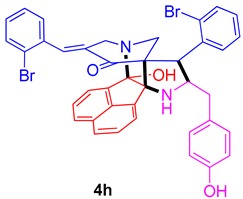	75.5 ± 3.8	68.4 ± 4	62.5 ± 4.6	44.9 ± 3.2
9	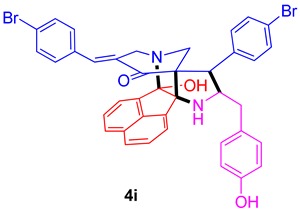	63.6 ± 4.2	69.7 ± 3.7	52.8 ± 2.4	61.3 ± 3.5
10	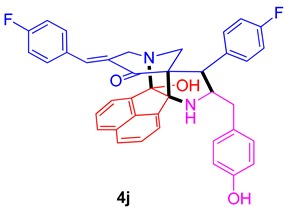	77.3 ± 3.8	82.7 ± 2.9	66.4 ± 3.8	58 ± 3.8
11	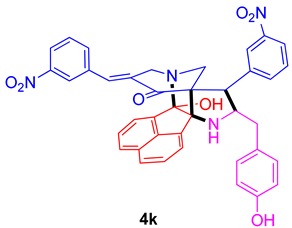	50.5 ± 3.7	50.2 ± 8.2	31.9 ± 4.9	35.3 ± 2.1
12	CPT	40.55 ± 2.4	38.5 ± 3.1	34.1 ± 3.6	31.5 ± 4.7

## Supplementary Materials

Electronic supplementary information can be accessed online.
