# Supplementary material for: Design, Synthesis and In Vitro Mechanistic Investigation of Novel Hexacyclic Cage-Like Hybrid Heterocycles
Source: Molecules. 2019 Oct 23;24(21):3820. doi: 10.3390/molecules24213820 (PMC6864531; doi:10.3390/molecules24213820)
Supplement: Supplementary file 1 [file molecules-24-03820-s001.pdf]

molecules

Article

Design, Synthesis, and In vitro Mechanistic Investigation of Novel Hexacyclic Cage-Like Hybrid Heterocycles

Raju Suresh Kumar [1,*], Abdulrahman I. Almansour [1], Natarajan Arumugam [1] and Faruq Mohammad[2]

[1] Department of Chemistry, College of Science, King Saud University, P.O. Box 2455, Riyadh 11451, Saudi Arabia; almansor@ksu.edu.sa (A.I.A.); anatarajan@ksu.edu.sa (N.A.)
[2] Surfactants Research Chair, Department of Chemistry, College of Science, King Saud University, P.O. Box 2455, Riyadh 11451, Saudi Arabia; fmohammad@ksu.edu.sa
* Correspondence: sraju@ksu.edu.sa or drrajusureshkumar@gmail.com

Supplementary data

Materials and Methods

3.1. Chemistry

General Methods

All other reagents and solvents were procured from commercial traders and used without further purification. Reaction progress was monitored by thin-layer chromatography on silica gel. Melting points were documented using open capillary tubes and are uncorrected. The ^{1}H, ^{13}C and 2D nuclear magnetic resonance (NMR) spectra were recorded on a Jeol 500 MHz instruments in $CDCl_3$ using TMS as internal standard. Standard Jeol software (Tokyo, Japan) was used throughout. Chemical shifts are given in parts per million (δ-scale) and the coupling constants are given in Hertz. Infrared (IR) spectra were recorded on a Perkin Elmer system 2000 Fourier transform infrared (FTIR) instrument (KBr) (Shelton, Alabama, USA). Mass spectra were recorded on a Triple quadrupole mass spectrometer (Micromass, Milford, CT, USA), Quattro Premier equipped with an electrospray ionization source (Z–spray) coupled with an Acquity ultra performance liquid chromatography (UPLC) system. Elemental analyses were performed on a Perkin Elmer 2400 Series II Elemental Carbon Hydrogen Nitrogen Sulphur (CHNS) analyzer (Waltham, MA, USA).

3.2. Biology

Dulbecco's phosphate buffered saline (DPBS), Fetal bovine serum (FBS), cell culture mediums like RPMI-1640, F-12K, DMEM (Dulbecco's modified eagle's medium), smooth muscle cell medium were obtained from HiMedia (Mumbai, Maharashtra, India). All the assay kits like Fluorescein isothiocyanate (FITC) rabbit anti-active caspase-3, FITC annexin V apoptosis detection kit I, Propidium iodide (PI), APO-Direct™ kit, MitoScreen JC-1 kit were obtained from BD Biosciences (San Jose, CA, USA). The CPT compound was purchased from Sigma-Aldrich (St. Louis, MO, USA). Human anti-caspase-3 antibody and cell culture slide hybrid wells were purchased from Life cell technologies (Bangalore, Karnataka, India).

3.2.1. Preparation of Stock Solutions

For the treatment, the testing compounds at different concentrations (5, 25, 50, 75, and 100 μM) and a positive control of camptothecin (CPT) (40 μM) were prepared in the dimethyl sulfoxide media. Further dilutions to the required concentrations of the testing compounds were made in either of F-

12K (A549 cells), RPMI-1640 (Jurkat cells), DMEM (MCF10 cells), or primary smooth muscle cell medium (PCS-130-010 cells) in order to treat during the in vitro molecular biology studies.

3.2.2. Cell Culture and Maintenance

For the investigation of in vitro biological response of the synthesized compounds labelled in the range of **4**(**a**–**k**), different assays such as cell viability and proliferation, apoptotic assay, caspase-dependent pathway, and so forth, were performed. For the testing, we have selected two cancerous (A549 cells of human adenocarcinoma alveolar basal epithelial origin and Jurkat cells of human acute T-cell lymphoma) and two non-cancerous cell lines (MCF-10 normal breast cell line and PCS-130-010 human primary lung smooth muscle cells) where they all obtained from the National Centre for Cell Science (NCCS, Pune, Maharashtra, India). In order to culture the cells, we used the respective mediums consisting of 10% FBS, penicillin (100 I.U./mL), streptomycin (100 μg/mL) at 37 °C in a humidified atmosphere of 5% CO_2 in air. For the testing, each time when the cell growth reaches to 80–90% confluency, the cells were separated out by tripsinization, counted, and transferred to a 96-well plate containing the complete growth medium at a density of 2×10^4 cells/well. Now the cells are allowed to adhere for at least 8 h before the final treatment and further, the samples are added to the cells containing 10% FBS of respective mediums.

3.2.3. Cell Viability and Proliferation Assay

The applied cell viability and proliferation assay for the testing of the synthesized compounds is based on the reduction of MTT agent, that is, 3-(4,5-dimethylthiazol-2-yl)-2,5-diphenyltetrazolium bromide. For the assay, the cells seeded into the 96-well plate (2×10^4 cells/well) when reach to their confluence levels were treated with the synthesized compounds **4**(**a**–**k**) at different concentrations in the range of 5–100 μM and incubated further for 24 and 48 h period in an incubator. Following the period, the cell medium was replaced with a fresh medium and the assay was followed according to the manufacturer's instruction where the absorbance was recorded at 570 nm wavelength. In this, CPT (40 μM) was selected as the positive control and the cells undergone without any treatment was taken as the negative control. Further, the IC_{50} values (concentration needed for 50% loss of cell viability) for all the synthesized compounds and CPT were investigated at all the incubation times and cell lines.

3.2.4. Apoptosis Assay

The cell death mechanism of the synthesized compound-treated A549 cells was analyzed by the Annexin V/FITC apoptotic kit, where the fluorescence intensity exhibited by the FITC-conjugated Annexin-V and PI-treated cells were analyzed by making use of the flow cytometry. Briefly, the A549 cells at a density of 10^6 cells/well were seeded in a 6-well plate and when the cell growth reaches to its confluence levels, were treated with the testing compound (**4a**) at its IC_{50} concentration as investigated by the cell viability and proliferation assay. After the incubation period (either of 24 or 48 h), the cells were undergone trypsinization, centrifugation to obtain the pellet which was re-suspended gently in 100 μL of AnnexinV-FITC binding buffer, and then incubated further in 5 μL of AnnexinV-FITC in the dark for 10 min at 25 °C. Now the cells were subjected to centrifugation (2000 rpm for 5 min) and the obtained pellet was re-suspended in 500 μL of AnnexinV-FITC binding buffer and 5 μL of PI was added in an ice bath, followed by the flow cytometric analysis. The Cell Quest software was applied for the investigation and analysis of apoptotic cells.

3.2.5. Cell Cycle Analysis

The A549 cells at a density of 10^6 cells/well were first seeded into a six-well plate and following the incubation for 12 h, the cells were treated with the compound **4a** at IC_{50} concentrations in a serum-free media and incubated for additional 24 or 48 h. After the period, the cells were undergone trypsinization and centrifugation (1000× *g* for 10 min) to obtain the pellet, which was re-suspended in PBS, followed by the fixation with the addition of 70% ethanol at least for 2 h. Now the cells were

centrifuged (1000× *g* for 10 min) and the obtained pellet was re-suspended in PBS; after 1 min, the cells were centrifuged, pellet was suspended again in PI (1 mL) staining solution. After the incubation of cells with PI for 15 min, the cells were subjected to the flow cytometric analysis at an excitation and emission wavelengths of 488 nm and 670 nm respectively using the Cell Quest software (San Jose, CA, USA).

3.2.6. Caspase-3 Expression

In order to test the role of caspase-3 for the compound **4a** treated A549 cells, the cells were first treated with IC_{50} concentrations and incubated for either 24 or 48 h periods (similar to earlier assays). After the period, the cells were harvested and then fixed with the ice cold ethanol (70%), followed by the incubation for additional 30 min at −20 °C. Now the cells were washed twice with PBS and then treated with Caspase-3 FITC reagent (20 μL), followed by the incubation at room temperature in dark for 60 min. Following the incubation in dark, the cells were washed with PBS and re-suspended in 0.5 mL of PBS and then analyzed for caspase-3 expression by the EVOS™ FL Auto fluorescence microscopy (Thermo Scientific, India) where the Image J software was applied.

3.2.7. Statistical Analysis

The results of in vitro cell culture systems presented are the mean ± SD of 3–5 individual experiments where the statistical analysis was based on student's *t*-test. For the statistical analysis, the significant value represented by * ($p < 0.05$) and highly significant value represented by ** ($p < 0.01$) against the control ran using the Graphpad prism software version 6 (San Diego, CA, USA).

NMR and IR spectra of a representative compound

Figure captions

- **Figure S1.** ^{1}H NMR spectrum of **4b.**
- **Figure S2.** ^{13}C NMR spectrum of **4b**.
- **Figure S3.** Dept 135 NMR spectrum of **4b**.
- **Figure S4.** COSY NMR spectrum of **4b.**
- **Figure S5.** HMQC NMR spectrum of **4b.**
- **Figure S6.** HMBC NMR spectrum of **4b.**
- **Figure S7.** FT-IR spectrum of **4b.**

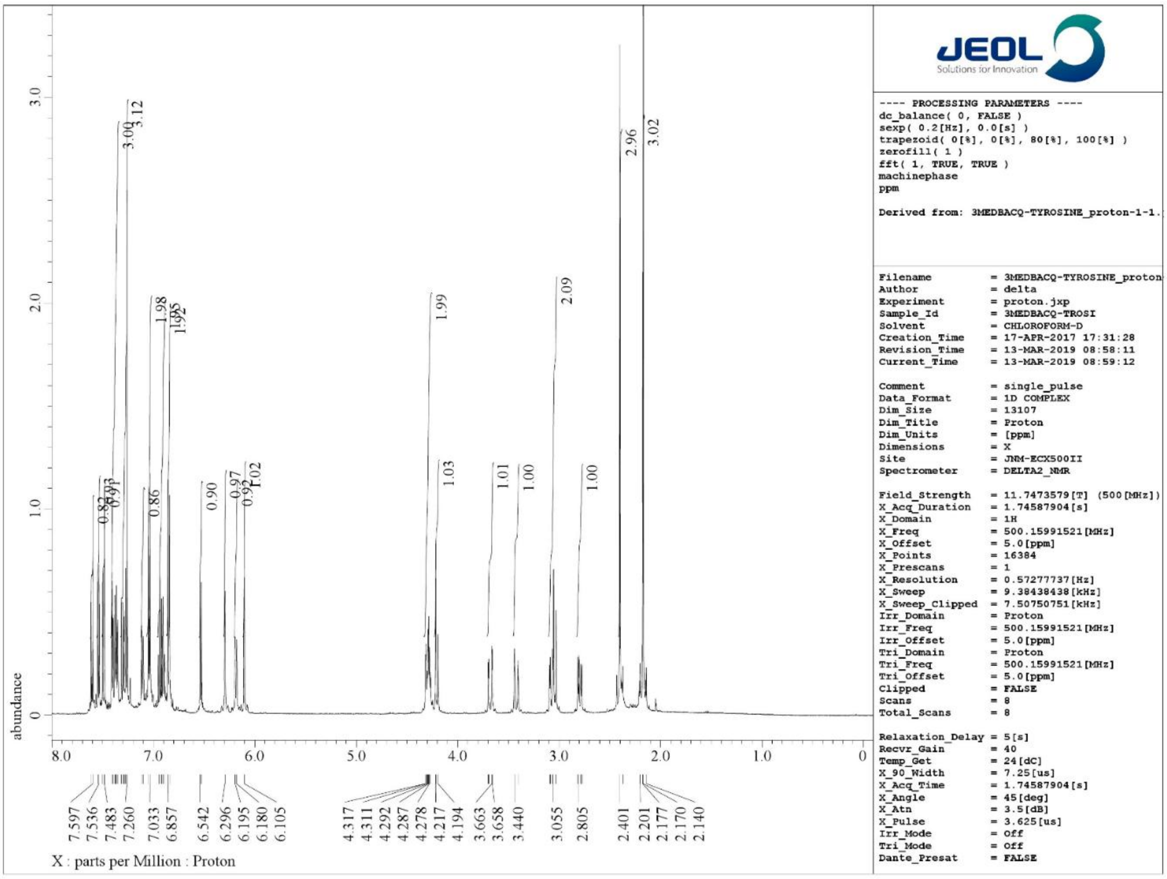

Figure S1. ^{1}H NMR spectrum of **4b.**

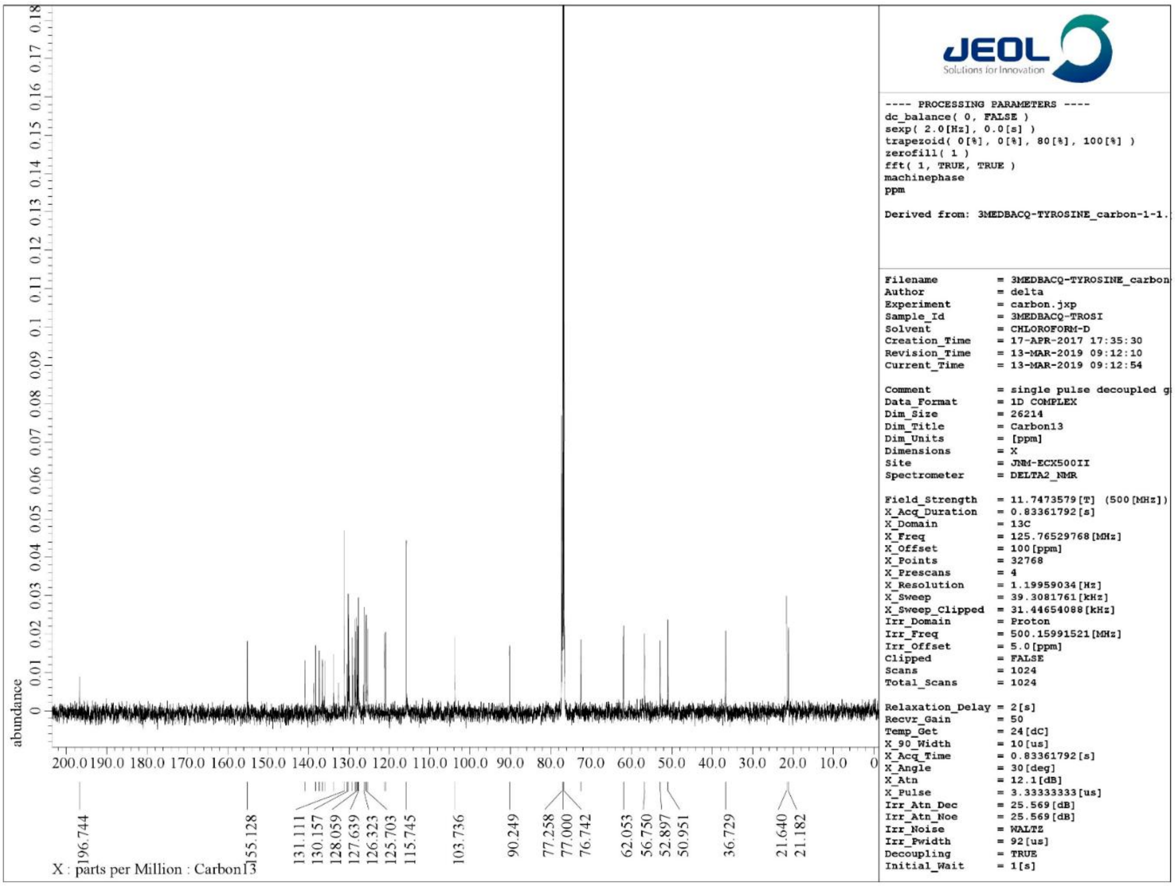

Figure S2. ^{13}C NMR spectrum of **4b.**

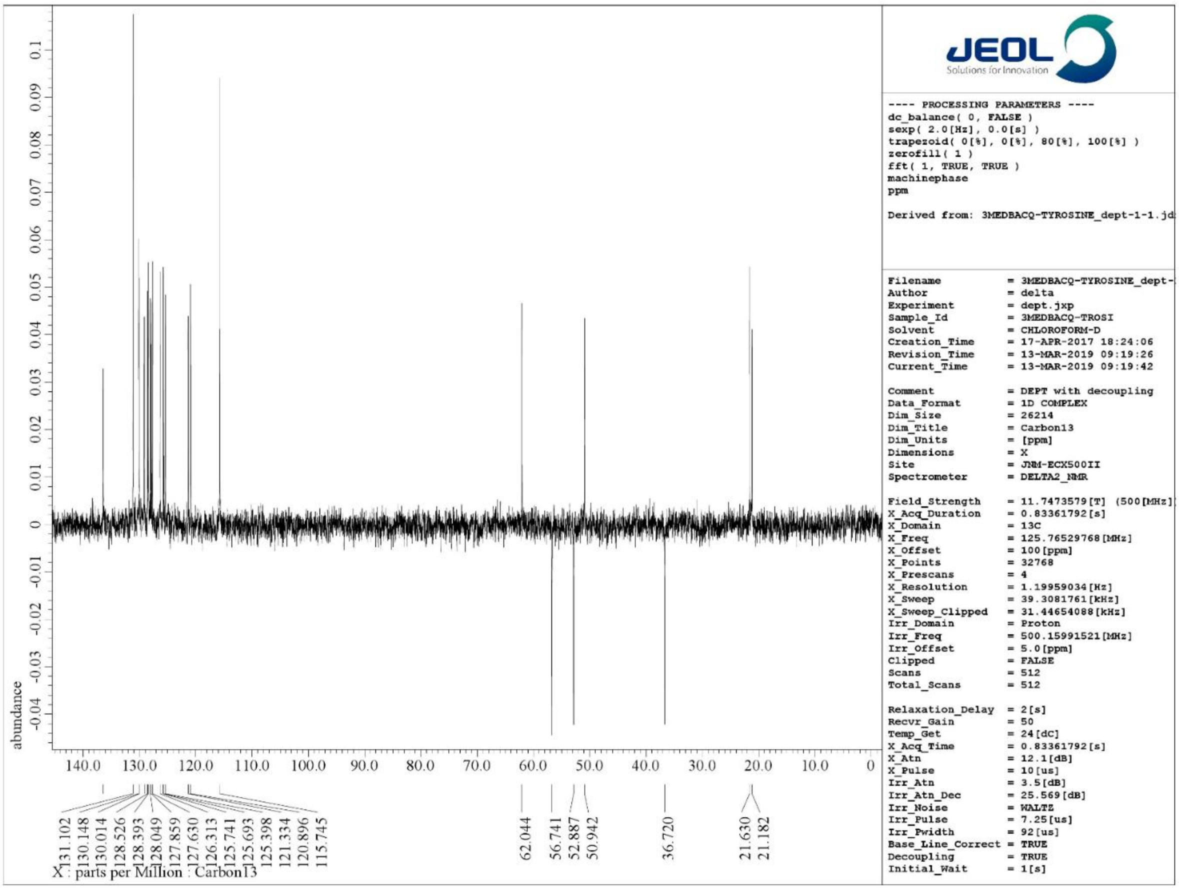

Figure S3. Dept 135 NMR spectrum of **4b.**

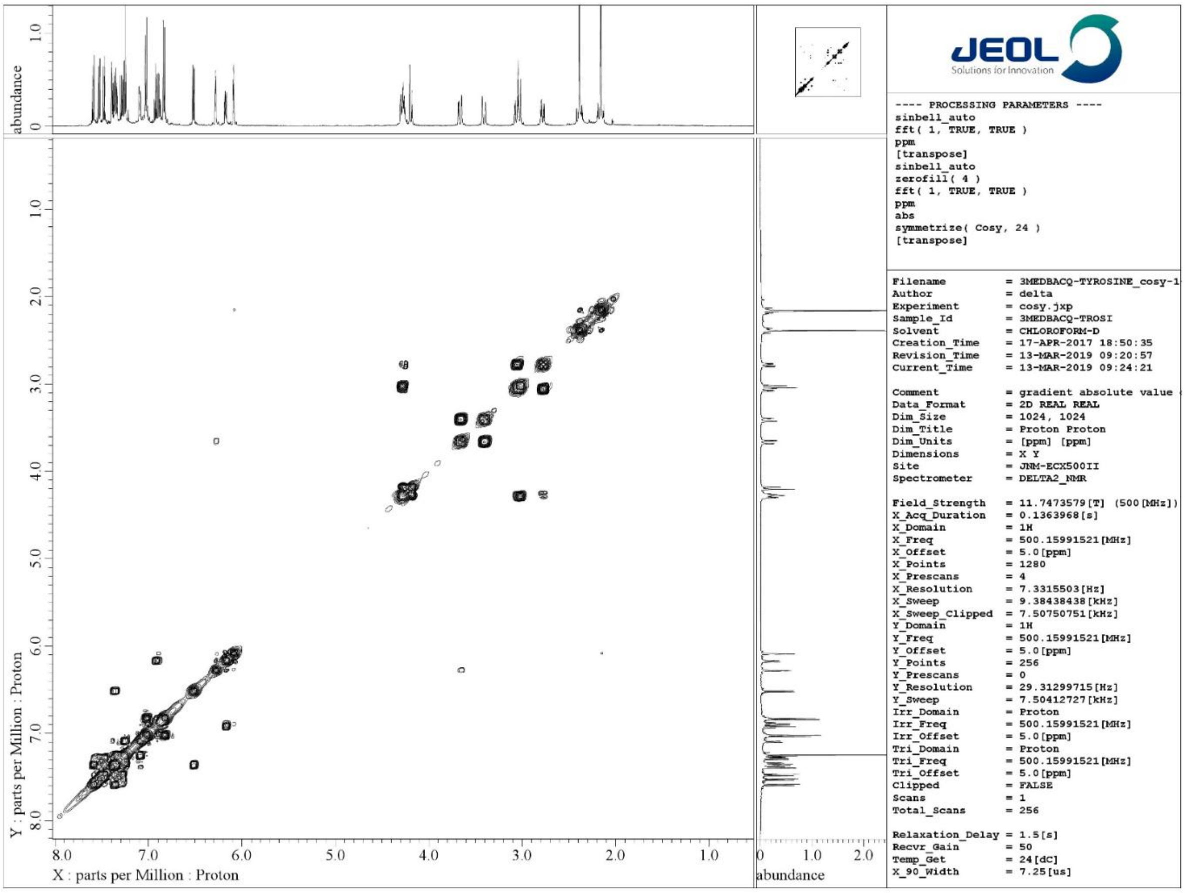

Figure S4. COSY NMR spectrum of **4b.**

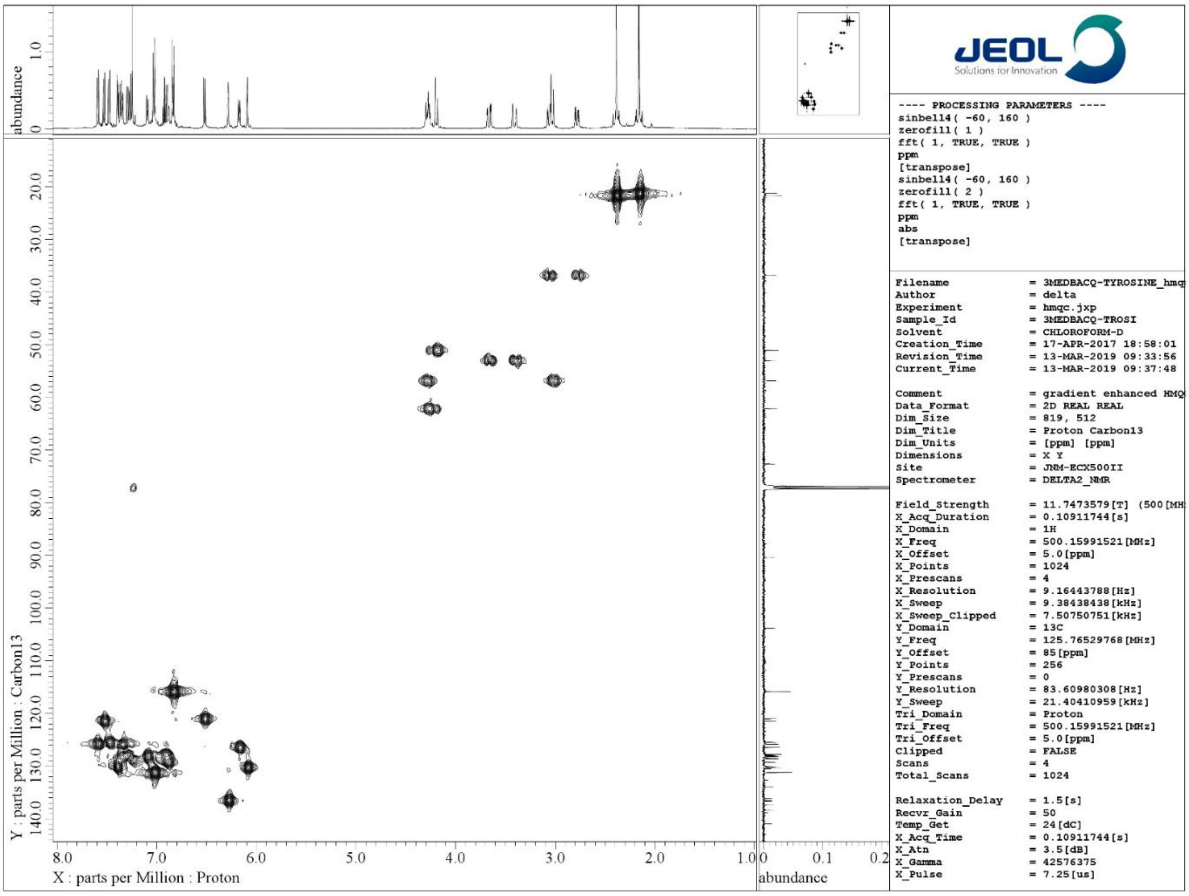

Figure S5. HMQC NMR spectrum of **4b.**

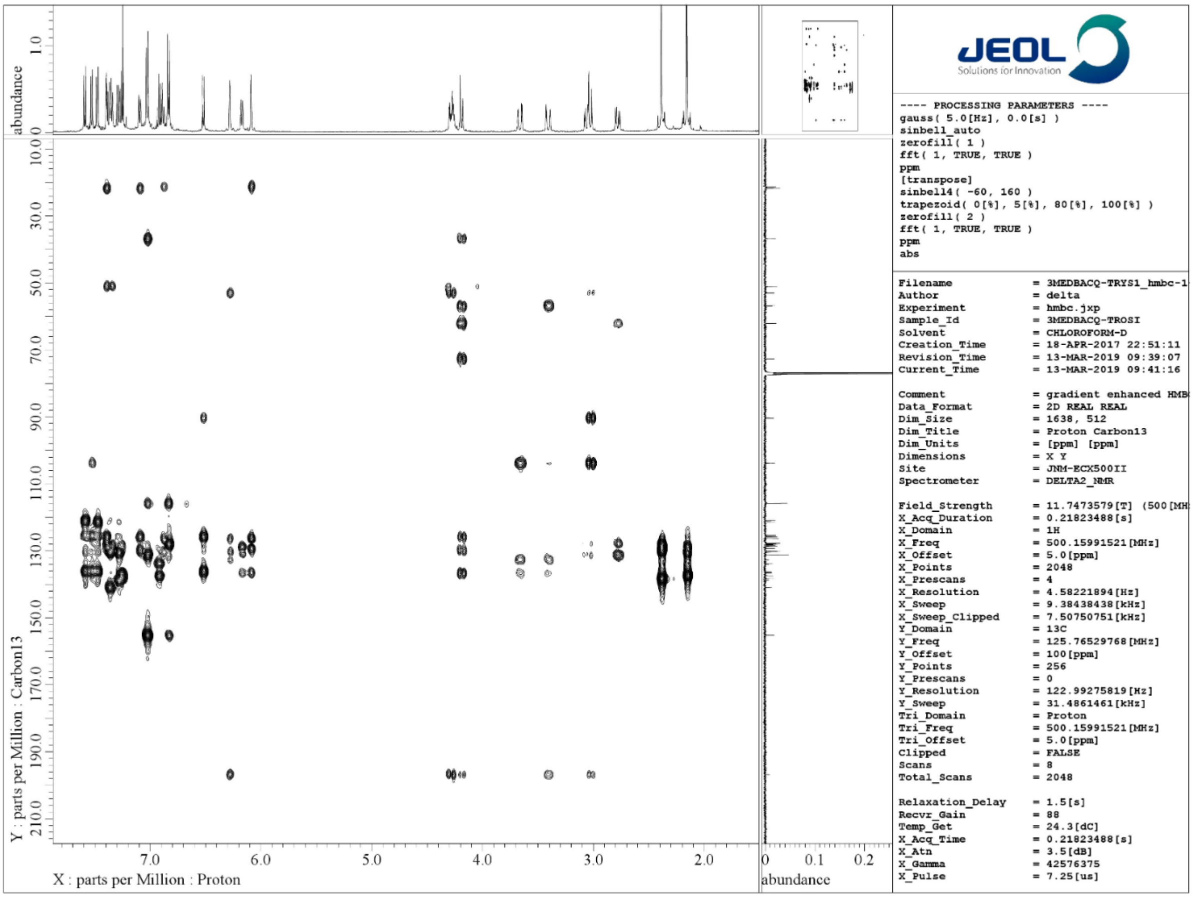

Figure S6. HMBC NMR spectrum of **4b.**

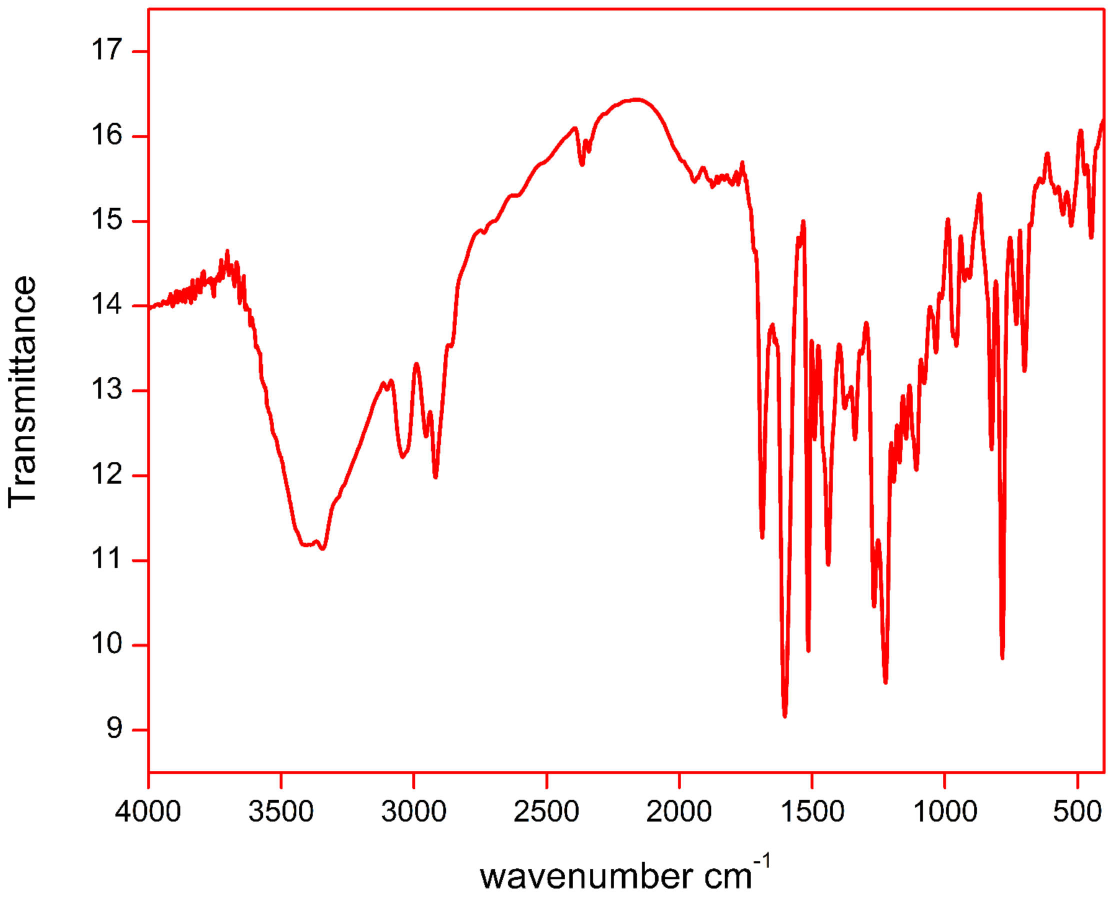

Figure S7. FT-IR spectrum of **4b.**
